# Determinants of physical activity among community-dwelling adults in Ghana

**DOI:** 10.4314/gmj.v60i1.6

**Published:** 2026-03

**Authors:** Hosea Boakye, Elizabeth Duah, Akua A Bilson, Martin Ackah, Thomas Hinneh, Ulric S Abonie, Albert Atabila

**Affiliations:** 1 Department of Biological, Environmental and Occupational Health, School of Public Health, University of Ghana, Accra, Ghana; 2 Department of Physical Therapy, Boston University, Boston, MA, USA; 3 Physiotherapy Department, LEKMA Hospital, Accra, Ghana; 4 Physiotherapy Department, The Trust Hospital Company Limited, Ghana; 5 Department of Sport, Exercise & Rehabilitation, Northumbria University, UK; 6 School of Nursing, Johns Hopkins University, Baltimore, Maryland, USA

**Keywords:** Physical activity, stages of behaviour change model, physical activity guidelines, sedentary behaviour

## Abstract

**Objectives:**

To examine the association between physical activity (PA) and knowledge of PA recommendations, sociodemographic, and behavioural characteristics among adults attending the outpatient department of a secondary health facility.

**Design:**

Cross-sectional using a multi-stage sampling method.

**Setting:**

Outpatient department of a secondary health facility.

**Participants:**

Adults aged 18 years and older attending the outpatient department of a secondary health facility.

**Main outcome Measures:**

Global Physical Activity Questionnaire (GPAQ)

**Results:**

Of 480 participants, the majority were females (57.7%), had a healthy weight (63.3%), were unmarried (44.6%), and had at least one child (58.5%). Less than half of the participants were in the pre-contemplation stage (30.8%), had adequate knowledge of physical activity recommendations (17.1%), and met recommended physical activity levels (46.3%). Females (Odds Ratio (OR) = 0.40, 95% Confidence Interval (CI) = 0.24-0.67, p < 0.001), older adults (OR = 0.23, 95% CI = 0.08-0.64, p = 0.005), people with high sedentary behaviour (OR = 0.47, 95%CI = 0.22-0.95, p = 0.036) and people in the pre-contemplation stage (reference) were less likely to engage in physical activity.

**Conclusions:**

Engaging in physical activity is strongly influenced by age, gender, sedentary behaviour, and stages of behaviour change. These determinants should be considered in interventions for promoting physical activity among community-dwelling adults.

**Funding:**

None declared

## Introduction

Globally, studies have consistently demonstrated the beneficial impact of physical activity (PA) on adult health. These benefits span multiple domains, including enhanced cardiovascular health, effective weight management, improved sleep quality, increased physical fitness, better cognitive function in later life, and reduced risk and favourable management outcomes for non-communicable diseases (NCDs).^1^–^7^ Consequently, the World Health Organisation (WHO) recommends that adults engage in at least 150 minutes of moderate-intensity, or 75 minutes of vigorous-intensity PA weekly.^8^ For effective uptake of these recommendations, countries have adopted the guidelines in the quest to design a culturally tailored and environmentally friendly intervention to promote PA.^9^ However, a significant portion of the global population does not meet the recommended PA guidelines^10^,^11^, with an estimated 27.5% of adults worldwide not meeting the recommendation for sufficient PA.^12^ Specifically, in Ghana, 11.2% to 29.0% of adults do not meet the recommended sufficient PA.^13^ This highlights the need to better understand predictors of PA to help guide the development of interventions to promote PA among adult populations.

An important variable that influences adults' participation in PA is their knowledge of established PA guidelines^14^, with the literature suggesting that individuals who meet the PA recommendations are more likely to be knowledgeable about them.^15^–^17^ Conversely, studies on knowledge and awareness of PA show that the general population's knowledge of the required amount of PA is very low, with only 2% and 3% of adults correctly identifying the recommended levels of moderate- and vigorous-intensity PA, respectively.^16^ Additionally, as many as 61% of adults who are not achieving the recommended level of PA overestimate their activity and are less likely to express an intention to change their behaviour.^18^ These findings are in line with the transtheoretical model, which posits that behaviour change starts with cognitive processes initiated by having adequate knowledge.^17^,^19^ This suggests that a lack of knowledge about PA may be an important barrier to behaviour change, and consequently result in individuals not recognizing a need to increase their PA and being unaffected by PA promotion efforts.^18^ Thus, when the nuances of individuals at different stages of behaviour change are identified, tailored interventions that might be more appropriate and effective could be implemented.

To date, most studies assessing knowledge of PA guidelines have been conducted outside Africa. Additionally, there is a dearth of data on the relationship between sociodemographic factors, PA knowledge, PA behaviour, and stages of change in Ghana. Given the notable differences in PA levels across countries, context-specific studies are crucial to understanding how PA relates to knowledge of PA guidelines at the national level.^20^ The findings that demographic characteristics are related to knowledge of PA guidelines^16^ coupled with paucity of studies in Ghana 21, underscore the need for population-based research to explore the relationship between PA levels, knowledge of PA recommendations, and stages of change. The current study aims to assess PA and explore its associations with knowledge of PA, socio-demographic characteristics, NCD, and behaviour change among community-dwelling Ghanaian adults.

## Methods

### Study design and setting

The study adopts a cross-sectional design conducted among adults attending clinics at a secondary health facility in Ghana.^22^

### Participants, recruitment, and sample size estimation

Participants were adults aged 18 and older attending the outpatient department at Ledzokuku Municipal Assembly (LEKMA) Hospital who consented to participate. Participants were recruited using a multi-stage sampling method: first, they were divided into three age groups (18–34, 35–49, and 50–64) based on 2021 data from the hospital's health information unit, with a ratio of 4.5:3.5:2. Simple random sampling was then applied, where participants drew slips from a container with ‘yes’ and ‘no’ options, with those selecting ‘yes’ being recruited into the study. Participants were excluded if they were wheelchair users, had cognitive impairments, were pregnant, or were currently undergoing supervised prescribed exercise. The sample size estimation is detailed in a previous study.^22^

### Study variables

#### Dependent variable

##### Physical activity

PA was measured using the Global Physical Activity Questionnaire (GPAQ) 23. The GPAQ has 16 questions on PA participation in three domains, viz. activity at work, travel to and from places, and recreational activities, with the last question on sedentary behaviour. The questionnaire was administered by trained research assistants. The data cleaning and scoring procedures were reported in accordance with established protocols. 23

Metabolic equivalents (METs) were used to analyse the data. Metabolic Equivalents for the individual activities were calculated, and the overall METs were obtained by adding all the individual METs. It is estimated that, compared to sitting quietly, a person's caloric consumption is four times as high when moderately active and eight times as high when vigorously active. Therefore, when calculating a participant's overall energy expenditure, 4 METs are assigned to time spent in moderate activities and 8 METs to time spent in vigorous activities. An accumulated MET of 600 MET minutes per week or more was set as the threshold for considering an individual physically active. 23

The psychometric properties of GPAQ have been evaluated in a systematic review, which reported moderate to good test–retest reliability, with correlation coefficients generally ranging from 0.50 to 0.80 across total and domain-specific physical activity.^24^ The review also found low to moderate concurrent validity when GPAQ outcomes were compared with objective measures such as accelerometers (correlations approximately 0.20–0.40), with higher correlations observed when compared with other self-report instruments.^24^

A pilot study of 15 participants was conducted to ascertain the feasibility of the proposed study. Based on the finding that the COVID-19 pandemic may influence PA participation, two extra questions were added to the study instrument to examine the impact of the COVID-19 pandemic on PA.

### Independent variables

#### Socio-demographic and anthropometric characteristics

The socio-demographic and anthropometric variables collected included age, gender, body mass index (classified as normal weight (18.5–24.9 kg/m^2^), overweight (25 – <30 kg/m^2^), and obese (≥30 kg/m^2^), marital status, educational level, occupation, and monthly income. Age, which was collected as a continuous variable and converted into a categorical variable (18–34 years, 35–49 years, 50–64years).

#### Non-Communicable Diseases

A single question was asked to determine if participants had been previously diagnosed with any NCD.

#### Knowledge of physical activity recommendations

Participants' knowledge of PA regarding dose recommendations was assessed using a self-administered questionnaire. Participants were asked to state the recommended PA level. Participants who correctly stated the recommended PA levels were scored as having knowledge of the guideline.

#### Behaviour change

Behaviour change was measured with the Physical Activity Stages of Behaviour Change questionnaire^25^, which was adopted to measure stages of PA behaviour. The questionnaire has four questions with Yes and No answers, which were coded and interpreted as pre-contemplation, contemplation, preparation, decision/action and maintenance stages. The questions are as follows: *“I am currently physically active”, “I intend to become more physically active in the next six months”, “I currently engage in regular physical activity” and “I have been regularly physically active for the past six months”*. The scoring and categorisation were performed based on established methods.^25^

#### Sedentary Behaviour

Sedentary behaviour was assessed with the last item of the GPAQ (P16). The time spent in sedentary behaviour during a typical day was recorded in minutes and dichotomised into 5 hours or more versus more than 5 hours.

#### Procedure for data collection

The data collection procedure is detailed elsewhere.^22^ Three research assistants (RAs), of whom two had a degree in rehabilitation, and one had a biochemistry degree, fluent in English and Ghanaian languages (Ga, Twi, Ewe), were recruited and underwent a two-day training workshop on infection prevention, COVID-19 safety protocols and questionnaire administration. The RAs practised the questionnaire under the researchers' supervision. Eligible participants were provided with an informed consent form, which they either signed or thumb-printed prior to participating and completing the questionnaires. Strict adherence to all COVID-19 protocols was maintained throughout the process.

### Ethical Approval

This study was approved by the Ghana Health Service Ethical Review Committee on Research involving Human Subjects (GHS-ECR 026/08/21). Permission was sought from the Management and Head of the OPD of the LEKMA Hospital. Written informed consent was obtained from study participants before enrolment. A detailed description has been reported^22^

### Statistical Analysis

A descriptive analysis was used to present the participants' demographic information. Frequencies, percentages, mean, and standard deviation (SD) were calculated, and the results were presented as distribution tables and bar graphs. A subgroup analysis was done to ascertain the relationship between gender and the different aspects of PA. Chi-square test of association was used to assess the association between the dependent variable and the independent variables. Statistically significant variables were selected for inclusion in multiple logistic regression models. Again, the chi-square test was used to examine the association between PA behaviour change stages and the presence of NCDs among adults. A Significance level was set at p < 0.05 with a 95% confidence interval. All statistical analyses were performed using Microsoft Excel and Stata version 16.

## Results

Five hundred eligible participants participated in the study. After cleaning the data, 20 were excluded due to missing or inaccurate data. Eventually, 480 participants were included in the final analysis, representing 96% of the total participants.

### Socio-demographic and anthropometric characteristics of the participants

Out of the 480 participants, 203 (42.3%) were males, and 277 (57.7%) were females. Participants' ages ranged from 18 to 64 years, with a mean of 37.7±16.5 years. The majority (304, 63.3%) had a normal BMI, while 176 (36.7%) were overweight or obese. Less than half, 185 (38.5%), of the participants were married, and a greater portion, 281 (58.5%), had at least one child. The majority, 311 (64.8%), had attained either primary or secondary education, while 139 (29%) had attained either technical/vocational or tertiary education, with 30 (6.3%) having no formal education. The majority, 218 (45.4%), were self-employed, followed by 100 (20.8%) who worked in the private-formal sector, with only 12 (2.5%) on retirement. The majority, 316 (65.8%), earn less than GHc 1000 ($173) per month, as presented in Table 1.

**Table 1:** Demographic, anthropometric and health characteristics of the participants

Variable	Frequency, N (%)
**Gender**	
**Male**	203(42.3)
**Female**	277(57.7)
**Age/years**	
**18-34**	223(46.4)
**35-49**	176(36.7)
**50-64**	81(18.9)
**BMI/Kg/m^2^**	
**Normal**	304(63.3)
**Overweight**	121(25.2)
**Obese**	55(11.5)
**Marital status**	
**Married**	185(38.5)
**Single**	214(44.6)
**Divorced**	17(3.5)
**Widowed**	20(4.2)
**Cohabitation**	44(9.2)
**Do you have a child**	
**Yes**	281(58.5)
**No**	199(41.5)
**Education**	
**No formal education**	30(6.3)
**Primary**	158(32.9)
**Secondary**	153(31.9)
**Technical/vocational**	56(11.7)
**Tertiary**	83(17.3)
**Occupation**	
**Unemployed**	74(15.4)
**Public sector**	37(7.7)
**Private sector**	100(20.8)
**Self-employed**	218(45.4)
**Student**	51(10.6)
**Monthly income [GHc]**	
**≤1000**	316(65.8)
**≥1000**	164(34.2)
**Non-communicable disease**	
**Yes**	128(26.7%)
**No**	352(73.3%)

### Participation in physical activity

Regarding PA participation, 222 participants (46.3%) met the recommended level, while 258 did not. A high proportion (425, 88.5%) did not engage in vigorous work-related activity. More males, 39(19.2%), engaged in vigorous work-related activity compared to females, 16(5.8%), as shown in Table 2. On average, participants spent 420.8±5.2 minutes per day engaged in sedentary activity. A higher proportion, 400 (83.3%), spent more than 300 minutes in sedentary activity, consisting of 254 (63.5%) females and 146 (36.5%) males.

**Table 2:** The categories of physical activity between males and females

Category of PA	Response	Gender		Total N(%)	p-value

		Male N(%)	Female N(%)		
**Vigorous activity at work**	Yes	39(19.2)	16(5.8)	55(11.5)	<0.001*
	No	164(80.8)	261(94.2)	425(88.5)	
**Moderate activity at work**	Yes	97(47.8)	102(36.8)	199(41.5)	0.016*
	No	106(52.2)	175(63.2)	281(58.5)	
**Total activity at work**	Yes	112(55.2)	113(40.8)	225(46.9)	0.002*
	No	91(44.8)	164(59.2)	255(53.1)	
**Travel**	Yes	41(20.2)	51(18.4)	92(19.2)	0.623
	No	162(79.8)	226(81.6)	388(80.8)	
**Vigorous Recreation**	Yes	76(37.4)	10(3.6)	86(17.9)	<0.001*
	No	127(63.6)	267(96.4)	394(82.1)	
**Moderate Recreation**	Yes	61(30.0)	74(26.7)	135(28.1)	0.422
	No	142(70.0)	203(73.3)	345(71.9)	
**Total Recreation**	Yes	115(56.7)	88(31.8)	203(42.3)	<0.001*
	No	77(43.3)	200(68.2)	277(57.7)	

* p<0.05

### Knowledge of physical activity recommendation

Eighty-two participants (17.1%) correctly stated the recommended dosage of physical activity, while 398 (82.9%) had no knowledge about the recommended dosage of physical activity.

### Physical activity stages of behaviour change and presence of NCDs

Considering the PA stages of behaviour change, 148 (30.8%) of participants were in the pre-contemplation stage, 13 (2.7%) were in the contemplation stage, 122 (25.4%) were in the preparation stage, 104 (21.7%) were in the decision/action stage, and 93 (19.4%) were in the maintenance stage. The majority, 273 (58.9%), were in the pre-action stage, and 197 (41.1%) were in the action stage.

Results show that 26.7% of participants reported being previously diagnosed with NCD22. The majority of participants (54, 42.2%) who reported NCD were in the precontemplation stage, while only 12 (9.4%) were in the maintenance stage. Among those who did not report any NCDs, 94 (26.7%) were in the pre-contemplation stage, while 81 (23.0%) were in the maintenance stage, as shown in Table 3.

**Table 3:** The categories of physical activity between males and females

Stages of behaviour change	Non-Communicable Disease		Total N(%)	p-value

	Diagnosed N(%)	Not Diagnosed N(%)		
**Pre-contemplation**	54(42.2)	94(26.7)	148(30.8)	**0.001**
**Contemplation**	6(4.6)	7(2.0)	13(2.7)	
**Preparation**	33(25.8)	89(25.3)	122 (25.4)	
**Action**	23(18.0)	81(23.0)	93 (19.4)	
**Maintenance**	12(9.4)	81(23.0	197 (41.1)	

### Associations between physical activity and independent variables

Results of the Chi-Square test (Table 4) showed a significant association between participation in PA and gender (X^2^ =37.65, p< 0.001), age range (X^2^ = 36.34, p<0.001), marital status (X^2^ = 14.76, p=0.005), having a child (X^2^ = 9.94, p=0.002), level of education (X^2^ =24.68, p< 0.001), occupation (X^2^ = 17.32, p=0.019), sedentary behaviour (X^2^ =101.43, p<0.001), stages of behaviour change (X^2^ =178.97, p<0.001), impact of COVID-19 pandemic (X^2^ =4.72, p=0.030) and presence of NCDs (X^2^ =12.68, p< 0.001).

**Table 4:** Associations of physical activity with independent variables

Independent Variable	Participation in physical activity		Chi-square	p-value

	Physically active	Physically inactive		
**Gender**				
**Male**	127	76	37.65	<0.001*
**Female**	95	182		
**Age/years**				
**18-34**	128	95	36.34	<0.001*
**35-49**	79	97		
**50-64**	15	66		
**BMI/Kg/m^2^**				
**Normal**	139	165	1.01	0.60
**Overweight**	60	61		
**Obese**	23	32		
**Marital status**				
**Married**	78	107	14.76	0.005*
**Single**	115	99		
**Divorced**	6	11		
**Widowed**	3	17		
**Cohabitation**	20	24		
**Have a child**				
**Yes**	113	168	9.94	0.002*
**No**	109	90		
**Education**				
**No formal education**	14	16	24.68	<0.001*
**Primary**	15	23		
**Middle**	40	80		
**Secondary**	68	85		
**Technical/vocational**	29	27		
**Tertiary**	56	27		
**Occupation**				
**Unemployed**	23	51	17.32	0.019*
**Public sector**	17	20		
**Private sector**	50	50		
**Self-employed**	101	117		
**Student**	31	20		
**Monthly income [GHc]**				
**≤1000($174)**	26	41	0.99	0.320
**≥1000($174)**	2	0		
**Knowledge of recommended physical activity**				
**Yes**	61	21	31.50	<0.001*
**No**	161	237		
**Stages of Change**				
**Pre-contemplation**	16	132	178.97	<0.001*
**Contemplation**	4	9		
**Preparation**	43	79		
**Decision/Action**	78	26		
**Maintenance**	81	12		
**Sedentary activity (Hours)**				
**≤5**	78	2	101.43	<0.001*
**≥5**	144	256		
**Impact of COVID-19**				
**Yes**	33	22	4.72	0.030*
**No**	189	236		
**Presence of NCD**				
**Yes**	42	180	12.67	<0.001*
**No**	86	172		

* p<0.05

There was no significant association between participation in PA and BMI and monthly income, as presented in Table 4.

### Determinants of physical activity

The results indicate that females are 60% less likely to engage in PA compared to their male counterparts. (Adjusted odds ratio (aOR) = 0.40, confidence interval (CI) = 0.24-0.67, p<0.001). Adults aged 50-64 years are 77% less likely to participate in PA than those aged 18-34 years (aOR=0.23, CI=0.08-0.64, P=0.006). Using the pre-contemplation stage as reference, the odds of an adult engaging in PA increase as one progresses from the pre-contemplation stage through the contemplation stage (AOR=4.46, CI=1.09-18.13, P=0.037), Preparation stage (AOR=3.25, CI=1.63-6.48, P<0.001), action stage (aOR=14.70, CI=6.94-31.13, P<0.001) and to the maintenance stage (aOR=35.26, CI=13.32-92.86, P<0.001). Adults who spend more than 5 hours in sedentary behaviour are 53% less likely to participate in PA than those who spend less than 5 hours in sedentary behaviour (AOR=0.47, CI=0.22-0.95, P<0.037). Marital status, education status, having a child, occupation, knowledge of PA guidelines, impact of the COVID-19 pandemic and the presence of NCDs did not independently predict participating in PA as shown in Table 5.

**Table 5:** Multiple logistic regression of physical activity determinants

	Adjusted Odd Ratio	95% Confidence Interval	p-value
**Gender**			
**Male**	Ref.		
**Female**	0.40	0.24-0.67	<0.001*
**Age/years**			
**18-34**	Ref.		
**35-49**	0.55	0.30-1.00	0.052
**50-64**	0.23	0.08-0.65	0.006*
**Marital status**			
**Married**	Ref.		
**Single**	0.72	0.37-1.41	0.340
**Divorced**	1.24	0.27-5.72	0.781
**Widowed**	0.50	0.10-2.42	0.390
**Cohabitation**	0.63	0.24-1.61	0.336
**Have a child**			
**Yes**	Ref.		
**No**	.81	0.43-1.52	0.511
**Education**			
**No formal education**	Ref.		
**Primary**	0.73	0.256-2.07	0.553
**Secondary**	0.77	0.27-2.23	0.633
**Technical/vocational**	0.58	0.17-1.97	0.385
**Tertiary**	0.90	0.25-3.22	0.868
**Occupation**			
**Unemployed**	Ref		
**Formal Public sector**	1.06	0.33-3.42	0.926
**Formal Private sector**	1.28	0.53-3.09	0.579
**Self-employed**	1.64	0.72-3.72	0.235
**Student/Apprenticeship**	1.88	0.64-5.50	0.250
**Knowledge of physical activity recommendation**			
**Yes**	Ref		
**No**	0.99	0.43-2.28	0.982
**Stages of change**			
**Pre-Contemplation**	Ref		
**Contemplation**	4.46	1.09-18.13	0.037*
**Preparation**	3.25	1.63-6.48	0.001*
**Action**	14.70	6.94-31.12	<0.001*
**Maintenance**	35.31	13.28-93.91	<0.001*
**Sedentary behaviour (hours)**			
**≤5**	Ref		
**≥5**	0.47	0.23-0.95	0.037*
**Impact of COVID-19**			
**Yes**	Ref		
**No**	0.64	0.27-1.52	0.316
**Presence of NCD**			
**Yes**	Ref		
**No**	0.91	0.51-1.88	0.948

* p<0.05

## Discussion

This study sought to assess PA participation and investigate its association with knowledge of PA recommendations, stages of behaviour change and sociodemographic characteristics among community-dwelling adults attending a secondary health facility in Ghana. The study found that more than half of the adults did not meet the recommended PA level. Additionally, knowledge of PA recommendations was very low, and most participants were in the pre-contemplation stage based on the transtheoretical model. Factors that independently influenced participation in PA were gender, age, stages of behaviour change and sedentary behaviour.

The study recorded very low (17.1%) knowledge of PA guidelines among Ghanaian adults. Previous studies have reported varying levels of knowledge across populations worldwide. Studies conducted in the United Kingdom^25^, the United States of America^27^,^28^, and Ethiopia^29^ reported higher knowledge levels than those reported in this study. On the contrary, a study conducted in Northern Ireland^30^ and China reported a lower knowledge level of PA guidelines. The disparities in the knowledge levels recorded could be attributed to the differences in information dissemination, lack of locally adopted and published PA guidelines, and differences in participants' sociodemographic characteristics such as gender, age, educational level, place of residence and income level.^29^–^33^

Regarding the PA level, findings from this study indicated that more than half (53.7%) of the participants did not meet the recommended PA level. Although there has been a reported decline in PA globally, the prevalence in this study is higher than reported in studies conducted in Ethiopia (46%)^34^ Nigeria (31.4%)^35^, Ghanaians residing in urban centers (29%) and Ghanaians residing in rural centers (11.2%)^13^ A benchmark study that included 358 studies from 168 countries with a population of 1.9 million also estimated that 27.5% of the world's adult population does not meet the recommended PA level.^36^ The higher prevalence recorded in the current study could be attributed to the impact of the COVID-19 pandemic. Measures to curb the spread of the virus, such as restrictions on movement (lockdowns), travel, and changes in work schedules (working from home), favoured physical inactivity. Although during the time of this study, the lockdown period in Ghana was over, people were still working from home, and some were having a shift system, such as two weeks at work and two weeks working from home. Similar findings have been reported by Mekanna et al.^37^. Additionally, the study by Afrifa-Anane^13^ utilised data from 2012 to 2015, which is almost a decade old. Therefore, changes in work conditions and transportation system in Ghana over the period that favors physical inactivity could attribute to the disparity.^38^

Regarding differences in gender and PA, this study revealed that more males than females were involved in vigorous recreation and vigorous and moderate activities at work, a difference that was statistically significant. A study that assessed gender differences in leisure-time PA from 1998 to 2018 found that more males engaged in high-intensity aerobic PA than females, whereas more females engaged in low-intensity aerobic PA than males.^39^ Mao et al. (40) indicated that more males than females were involved in competitive sports, aerobic exercises, strengthening exercises, and walking, while females were more involved in recreational activities than males. This was corroborated by a cross-sectional survey indicating that females were less likely to be involved in vigorous, competitive, outdoor activities that require skills and practice.^41^ This calls for gender-specific interventions to improve PA. Thus, assessing gender specific motivational factors, type of exercises preferred, and the barriers could help mitigate the low level of PA, especially among females. As Ghana transitions from a pre-industrial to an industrial state, where most vigorous and moderate activities at work will be performed mechanically, it behoves exercise specialists, health professionals, public health practitioners, and policymakers to implement policies and pragmatic PA behaviour change that focus on PA involving travel and recreation.

Findings from the univariable analysis indicated an association between PA and gender, age, marital status, having a child, education, occupation, sedentary behaviour, stage of behaviour change, and the impact of the COVID-19 pandemic. However, when these variables were subjected to multiple logistic regression, only gender, age, stages of behaviour change, and sedentary behaviour were found to be independent predictors of participation in PA.

To promote PA among adults globally, the determinants of PA are being studied to inform targeted interventions. Gender has been identified as a strong independent determinant of PA. This study indicates that females were 60% less likely to engage in PA than their male counterparts. Several previous studies have reported similar findings.^12^,^34^, ^35^ Culturally tailored and gender-based interventions based on preferences and motivations, while addressing the barriers, could help improve PA, especially in females.^9^,^39^,^14^

One interesting finding of this study is that the stages of PA behaviour change independently influenced PA participation. As one progresses from the pre-contemplation, contemplation, preparation, and action stages to the maintenance stage, the odds of engaging in PA increase. It is worth noting that as one moves from the preparation stage to the action stage, the odds of engaging in PA increase by more than 4 times. The plausible explanation is that, at the preparation stage, the individual has gained knowledge about PA and is preparing to engage in the activity, but has not yet initiated the action.^41^ Whereas, at the action stage, the individual will have engaged in PA for less than 6 months. At the maintenance stage, the odds of participating in PA were about 2.5 times that of the action stage. This is because at the maintenance stage, the individual would have engaged in PA for more than 6 months and therefore relapse was less likely to occur than the action stage. Noteworthy is that, except for the preparation stage, which had a similar percentage of adults with or without NCDs, the percentage of adults with NCDs decreased from the pre-contemplation stage through the maintenance stage, while the percentage of the adults without NCDs increased from the pre-contemplation stage. Different strategies of health promotion education have been suggested for individuals at the different stages of the transtheoretical model. For example, highlighting the risk of physical inactivity and addressing common misconception would be ideal health promotion strategy for individuals in the precontemplation stage while reinforcing the benefits achieved since participating in PA and providing measures to avoid relapse will be ideal strategies for individuals in the maintenance stage^19^. Based on the results of this study, health promotion strategies in improving and maintaining PA should differ from adults with and without NCDs. Thus, health professionals and public health specialists should provide targeted PA behaviour change intervention for people assessing healthcare.

Additionally, our study revealed that engaging in sedentary behaviour for more than 5 hours a day decreases the odds of engaging in PA. This finding is corroborated by a systematic review by Mansoubi et al. 43 which indicated that all forms of sedentary behaviour are inversely associated with PA and displace light PA. Therefore, interventions aimed at decreasing sedentary behaviour will increase PA, especially light intensity PA.

### Clinical implication and relevance

This study provides clinically relevant evidence on the determinants of physical activity among community-dwelling adults in Ghana, highlighting critical gaps in knowledge of physical activity recommendations and low engagement in adequate physical activity. The findings underscore the importance of incorporating physical activity education and behaviour-change counselling into routine clinical and primary healthcare services, particularly for women, older adults, and individuals with high sedentary behaviour, who were less likely to meet recommended activity levels. By identifying modifiable behavioural and knowledge-based factors associated with physical inactivity, this study supports the use of stage-based and targeted interventions, such as those guided by the Transtheoretical Model, to promote physical activity and prevent non-communicable diseases. These results have important implications for clinicians, public health practitioners, and policymakers aiming to strengthen preventive care and lifestyle interventions in Ghanaian settings.

### Strengths and limitations

This study is crucial for developing tailored behaviour change interventions to improve PA among adults. Additionally, the diverse background of the participants and the sample size have implications for the generalizability of the results. However, the study has some limitations. We relied on self-reported PA, which could introduce recall bias. Nevertheless, the use of the GPAQ, which has been validated, may help mitigate this bias. Furthermore, the cross-sectional design of the study limits the ability to establish causality between PA and the observed determinants.

## Conclusion

A significantly large proportion of adults do not meet the recommended PA levels. Other findings included a notable lack of knowledge regarding PA recommendations. Furthermore, the majority of participants were found to be in the pre-action stage according to the transtheoretical model of behaviour change. Gender, stages of behaviour change, and sedentary behaviour were identified as factors associated with participation in PA among adults in Ghana. These highlight the need for context-specific interventions to increase knowledge of PA recommendations and behaviour change, thereby promoting PA participation among the studied population.
